# Saikosaponin A Alleviates Symptoms of Attention Deficit Hyperactivity Disorder through Downregulation of DAT and Enhancing BDNF Expression in Spontaneous Hypertensive Rats

**DOI:** 10.1155/2017/2695903

**Published:** 2017-02-15

**Authors:** Sun Jichao, Han Xinmin, Ren Xianguo, Yin Dongqi, Zhou Rongyi, Lei Shuang, You Yue, Song Yuchen, Ying Jingnan

**Affiliations:** ^1^The First Clinical College, Nanjing University of Chinese Medicine, Nanjing, Jiangsu 210023, China; ^2^Jiangsu Key Laboratory of Pediatric Respiratory Disease, Institute of Pediatrics, Nanjing University of Chinese Medicine, Nanjing, Jiangsu 210023, China; ^3^Nanjing General Hospital of Nanjing Military Command, Nanjing, Jiangsu 210002, China; ^4^Affiliated Hospital of Nanjing University of Chinese Medicine, Nanjing, Jiangsu 210023, China

## Abstract

The disturbed dopamine availability and brain-derived neurotrophic factor (BDNF) expression are due in part to be associated with attention deficit hyperactivity disorder (ADHD). In this study, we investigated the therapeutical effect of saikosaponin a (SSa) isolated from* Bupleurum Chinese* DC, against spontaneously hypertensive rat (SHR) model of ADHD. Methylphenidate and SSa were orally administered for 3 weeks. Activity was assessed by open-field test and Morris water maze test. Dopamine (DA) and BDNF were determined in specific brain regions. The mRNA or protein expression of tyrosine hydroxylase (TH), dopamine transporter (DAT), and vesicles monoamine transporter (VMAT) was also studied. Both MPH and SSa reduced hyperactivity and improved the spatial learning memory deficit in SHRs. An increased DA concentration in the prefrontal cortex (PFC) and striatum was also observed after treating with the SSa. The increased DA concentration may partially be attributed to the decreased mRNA and protein expression of DAT in PFC while SSa exhibited no significant effects on the mRNA expression of TH and VMAT in PFC of SHRs. In addition, BDNF expression in SHRs was also increased after treating with SSa or MPH. The obtained result suggested that SSa may be a potential drug for treating ADHD.

## 1. Introduction

Attention deficit hyperactivity disorder (ADHD) is a common childhood neurodevelopment disorder characterized by a persistent of inattention, hyperactivity, and/or increased impulsivity, pervasive across setting, and this may lead to various degrees of functional impairment [1]. Epidemiological studies indicate that ADHD can affect 5–10% of children and up to 5% adults [2]. According to the Diagnostic and Statistical Manual (DSM-5), three subtypes have been identified: predominantly hyperactivity/impulsive type, predominantly inattentive type, and combined type. It is a major clinical and public health problem as individuals suffering ADHD have much higher incidence of cocaine abuse when comparing with age matched healthy individuals [3, 4].

Despite the high incidence of ADHD, the exact pathophysiology of ADHD remains unclear [5]. Previous work revealed that no single risk factor is either necessary or sufficient to explain ADHD, which indicated that many genetic and nongenetic (or environment) factors, including genetic, heredity, gene-environment interplay, and environment, may be involved in the occurrence and development of the ADHD [6–9]. It is widely accepted that dysfunction of catecholamine and particular dopamine (DA) neuronal systems (dopaminergic hypothesis) plays an important role in the pathophysiology of ADHD [10, 11]. DA is associated with attentional, reinforcing, psychomotor, and rewarding behavior, and DA deficits in cortical areas and/or striatum could induce obvious ADHD symptoms. DA synthesis, vesicular localization and release, and extracellular persistence were dynamically regulated by several factors, including activity of tyrosine hydroxylase (TH), vesicles monoamine transporter (VMAT), and dopamine transporter (DAT) [12, 13]. TH was the rate-limiting enzyme involved in the converting L-tyrosine to L-dihydroxyphenylalanine (L-DOPA), which ultimately converted to DA. DA was then transported from cytoplasmic space into synaptic vesicles by VMAT2 expressed in presynaptic terminals. The released DA could active the DA receptor in both presynaptic and postsynaptic membrane. The DA would be taken back to dopaminergic terminals by DAT in the presynaptic membrane and then degraded by monoamine oxidase. All these factors may ultimately disturb the metabolism, transporting and utilizing the DA. Brain-derived neurotrophic factor (BDNF) could affect dopaminergic neurotransmitter systems and be also involved in the learning and memory efficiency. Recent work has revealed that genetic variation of BDNF or dysregulated BDNF/tropomyosin-related kinase B (TrkB) pathway may impair learning and memory performance in ADHD childhood or animal models [14, 15].

As a worldwide first-line treatment drug, the efficacy of methylphenidate against ADHD has been empirically recognized for decades. MPH gets its pharmacological effect by modifying the DA system; it can block the DAT and noradrenaline (NA) transporters and then increase the extracellular DA concentrations in the prefrontal cortex and striatum of ADHD children or experimental animals [16, 17]. However, the therapeutic effects only manifested in 70%–80% of ADHD patients. Therefore, alternative therapeutic strategy for ADHD was urgently demanded and aroused substantial interest.

Saikosaponin a (SSa) is the primary triterpene saponin isolated from* Bupleurum Chinese *DC (Umbelliferae) [18] with various pharmacological effects, including anti-inflammatory, immunomodulatory, and bacterial activities [19]. Furthermore, SSa has been used to treat neurologic disease, such as epilepsy [20], neuropathic pain [21, 22], and Alzheimer's disease [23]. The relevant researches illuminate SSa has the pharmacological function of antineuroinflammation and attenuation of neuropathic pain. However, its pharmacological activity on neurodevelopment diseases, such as ADHD, has not been previously studied. The current study was initiated to investigate the possible pharmacological effect of SSa in terms of DA system, which is responsible for the ADHD pathologies and its underlying mechanisms.

## 2. Materials and Methods

### 2.1. Reagents

SSa was purchased from MUSTBio-Technology Co. (Chengdu, China) and methylphenidate was obtained from Suzhou No. 1 Pharmaceutical Co. (Suzhou, China).

### 2.2. Experimental Animals

Male spontaneously hypertensive rats (SHRs) (*n* = 50) and 10 Wistar-Kyoto (WKY) rats (*n* = 10), weighing 64–72 g, were purchased from Beijing Vital River Laboratory Animal Technology Co. Ltd. (Beijing, China). The rats were kept in a controlled environment with temperature set at 23 ± 1°C and the relative humidity was 50 ± 5% with 12 h light/darkness cycle. All animals were with free access to food and water and the animal studies were conducted in accordance with protocols approved by the Animal Ethic Committee of Nanjing University of Chinese Medicine.

### 2.3. Drug Treatment

WKY rats served as control (*n* = 10), and SHRs were randomly divided into 5 groups with 10 rats in each group: model group (untreated SHRs), methylphenidate (MPH) treated (1.5 mg/kg) group, the 12.5 mg/kg saikosaponin a-treated group, the 25.0 mg/kg saikosaponin a-treated group, and the 50.0 mg/kg saikosaponin a-treated group. MPH group were orally given MPH at a dose of 1.5 mg/kg [24], while saikosaponin a groups were orally given saikosaponin a in doses of 12.5, 25.0, and 50.0 mg/kg, once daily for 3 weeks. Rats in control group (WKY rats) and model group received an equal amount of saline.

### 2.4. Behavioral Testing

#### 2.4.1. Open-Field Test

Previous work revealed that SHRs exhibited an increased ambulatory and rearing activity which was similar to hyperactivity of children with ADHD. After treating test drugs, rats were subjected to the open-field test. The open-field board consisted of an iron box (90 cm × 90 cm × 50 cm) and was divided into 25 squares of 18 cm × 18 cm (16 peripheral squares and 9 central squares). A 60-watt light was situated 1 meter above the arena floor. Before each trial, the floor was cleaned thoroughly with 70% ethanol. Rats were placed in the center square of the floor for 30 seconds and covered with an opaque black casket. After removing the casket, the number of squares crossed (horizontal activity), rearing frequency (vertical activity, defined as number of times the animals stood on their hind legs), and moving distance were recorded for analysis of locomotor activity in a testing period of 5 minutes.

#### 2.4.2. Morris Water Maze Test

To assess spatial learning and memory, rats (*n* = 10/group) were tested in the Morris water maze. The Morris water maze (Beijing Shuolinyuan Science and Technology Co., Ltd., Beijing, China) is composed of the monitor with the video camera set in the ceiling, a computerized tracking system, and a black circular metal tank (150 cm in diameter, 60 cm in height) filled with water (24 ± 2°C). A transparent plastic platform (10 cm in diameter, 45 cm in height) was located at the center of a fixed quadrant and submerged about 1 cm below the water surface. Training started after the rat was acclimating to the task environment with 1 day of free-swimming in the pool with no platform. Each session lasted for 2 minutes, after which the rat was removed from the pool. The rats then received training of 4 trials per day for 4 consecutive days. The intervals between trials were 60 seconds. In each trial, the rat was placed into the water randomly in one of the four quadrants. The rats then had to swim until they climbed onto the platform submerged underneath the water. The duration from the time when the rats entered the water to the time when they climbed onto the platform was recorded and defined as the escape latency. Swimming distance was also recorded. If the rat failed to find the platform by 120 seconds, it was taken on the platform by the experimenter for 60 seconds. At the end of each day's training, the rat was dried before placed into home cage.

### 2.5. Determination of DA in Prefrontal Cortex and Striatum

Rat prefrontal cortex and striatum (about 20 mg) were weighed and sonicated in 100 *μ*L of a 0.1 M HClO_4_/10 *μ*M ascorbate solution for 1 min; brain samples were then centrifuged at 12,000*g* for 10 min at 4°C. For the determination of DA, samples were eluted with a mobile phase containing 25 mM acetate buffer with 0.75 mM sodium heptanesulfonate (pH 3.9) and methanol (85 : 15, v/v) on a Hypersil ODS column (250 mm × 4.6 mm, 5 *μ*m) with flow rate set at 1.0 mL/min. An aliquot of 20 *μ*L of supernatant was injected to a high performance liquid chromatography (HPLC) system equipped with a fluorescence detector (Waters, USA). The excitation and emission wavelengths were set at 305 and 360 nm, respectively.

### 2.6. Real Time-PCR

Total RNA from prefrontal cortex samples was extracted using Trizol reagent (Takara Biotechnology, Dalian, China) according to the manufacturer's instructions. RNA was reverse transcribed to cDNA using a Takara PrimeScript 1st Strand cDNA Synthesis Kit and the obtained cDNA was used as a template to perform PCR amplification using SYBR® Premix Ex Taq™II kit (Takara Biotechnology, Dalian, China). The specific primers for TH were sense, 5′-AGCCTGTGTACTTTGTGTCCGAGA-3′ and antisense, 5′-TGTGAGGGCTGTCCAGTACGTC-3′; for VMAT2 were sense, 5′-CCTTCGAAGTCCACCTGCTAA-3′ and antisense, 5′-CATCACCGATGGGATATGACTG-3′; for DAT were sense, 5′-AGACACCAGTGGAGGCTCAAGA-3′ and antisense, 5′-GCATCCCAGCAATAACCATGAAG-3′; and for GAPDH were sense, 5′-GACATGCCGCCTGGAGAAAC-3′ and antisense, 5′-AGCCCAGGATGCCCTTTAGT-3′. Each 20 *μ*L reaction system consisted of 2 *μ*L cDNA, 10 *μ*L SYBR Premix Ex Taq II, and 10 *μ*mol/L of both sense and antisense primers. Three replicates were performed for each quantitative PCR run. The mRNA concentrations of all target genes were normalized to that of the GAPDH in each sample (using delta-delta C_t_ method).

### 2.7. Western Blot

The proteins were extracted from prefrontal cortex tissues using RIPA lysis buffer (Beyotime, Nantong, China) according to the operating instructions. The protein concentration in the lysates was evaluated using a BCA protein assay kit (Thermo Fisher Scientific, MA, USA). The protein samples were separated on a SDS-PAGE and transferred to a polyvinylidene difluoride membrane (Bio-Rad, CA, USA). Membranes were blocked with 5% fat milk in TBS containing 0.1% Tween 20, followed by incubating with VMAT2 antibody (ab70808, 1:750), DAT antibody (ab5666, 1:1000), and BDNF antibody (ab108319, 1:2000) (Abcam, MA, USA) overnight at 4°C. Then, the blots were washed and incubated with horseradish peroxidase-conjugated secondary antibody. Band detection was performed using the enhanced chemiluminescence (ECL) detection kit (Thermo Fisher Scientific, MA, USA). The detected bands were calculated densitometrically using Image-Pro® Plus 5.0 software (Media Cybernetics, Bethesda, MD, USA). Data were adjusted to corresponding *β*-actin expression to avoid possible variations of protein expression.

### 2.8. Statistical Analysis

All data were performed using the SPSS® software version 22.0 (IBM Inc., Somers, NY, USA). Data were expressed as the mean ± standard error (SE). Statistical differences among these three groups were evaluated using one-way analysis of variance (one-way ANOVA) followed by a LSD post hoc test. *p* value less than 0.05 was considered to be statistically significant.

## 3. Results

### 3.1. Effects of SSa on Locomotion in SHRs

**Figure 1:**
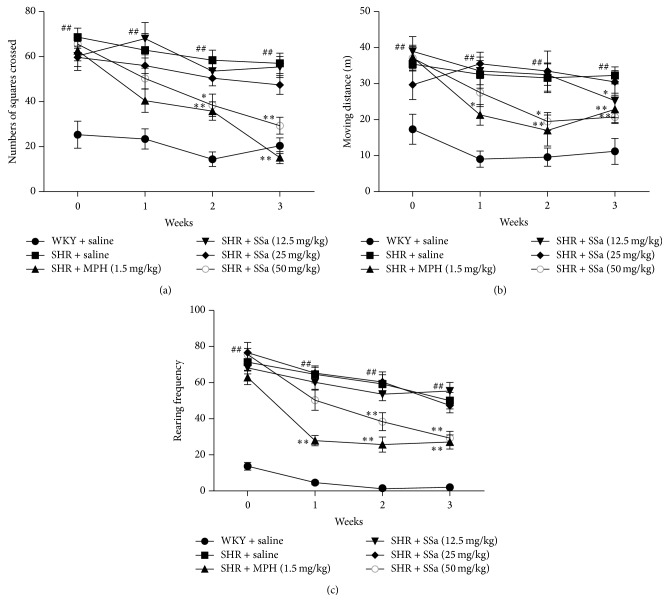
Open-field testing. (a), (b), and (c) represent horizontal activity, vertical activity, and locomoter behavior, respectively. Data are expressed as means ± SEM. *n* = 10, ^#^*p* < 0.05 and ^##^*p* < 0.01 versus WKY + saline group; ^*∗*^*p* < 0.05, ^*∗∗*^*p* < 0.01 versus SHR + saline group.

In accordance with the previous studies, SHRs were more active than WKY rats. As shown in Figure 1, SHRs showed increased numbers of square crossings (*p* < 0.01), moving distance (*p* < 0.01), and rearing (*p* < 0.01) when comparing with saline-treated WKY group before drug treatment. After treating test drugs one week later, moving distance (*p* < 0.05) and rearing (*p* < 0.01) in SHRs treated with MPH were reduced as compared to saline-treated SHRs. No significant difference was observed among SSa groups on square crossings as compared to that of saline-treated SHRs.

After treatment of these drugs for two weeks, SHRs treated with MPH and SSa at doses 50 mg/kg showed decrease in the numbers of square crossings (MPH, *p* < 0.01; 50 mg/kg, *p* < 0.05), moving distance (MPH, *p* < 0.01; 50 mg/kg, *p* < 0.05), and rearing (MPH, *p* < 0.01; 50 mg/kg, *p* < 0.01) as compared to saline-treated SHRs.

After drug therapy for three weeks, SHRs treated with MPH and SSa at doses of 50 mg/kg reduced the numbers of square crossings (MPH, *p* < 0.01; 50 mg/kg, *p* < 0.01), moving distance (MPH, *p* < 0.01; 25 mg/kg, *p* < 0.05; 50 mg/kg, *p* < 0.01), and rearing (MPH, *p* < 0.01; 50 mg/kg, *p* < 0.01) when comparing with saline-treated SHRs.

### 3.2. Effects on SSa on Morris Water Maze Test

**Figure 2:**
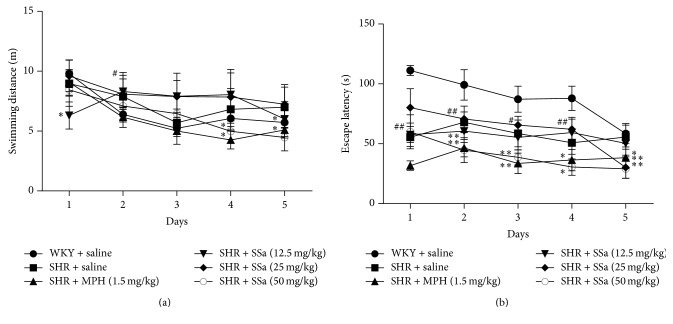
Morris water maze test. Escape latency (a) and swimming distance (b) represent rats' spatial learning. Data are expressed as means ± SEM. *n* = 10, ^#^*p* < 0.05 and ^##^*p* < 0.01 versus WKY + saline group; ^*∗*^*p* < 0.05, ^*∗∗*^*p* < 0.01 versus SHR + saline group.

As shown in Figure 2, the swimming distance (Figure 2(a)) and escape latencies (Figure 2(b)) decreased in these WKY rats or SHRs during the training period which reflected that these rats have intact spatial learning. During acquisition training, saline-treated WKY rats swam a longer escape latency to find the platform than SHRs except the last day (*p* < 0.01). After the first training day, SHRs treated with MPH and SSa at dose of 50 mg/kg swam a shorter escape latency to find the platform compared with saline-treated SHRs on the following training days (*p* < 0.05 or *p* < 0.01). SHRs treated with SSa at dose of 25 mg/kg exhibited a shorter escape latency to find the platform as compared to saline-treated SHRs on the last test day (*p* < 0.05). On the last two training days, SHRs with MPH and SSa at dose of 50 mg/kg had a shorter swimming distance compared with saline-treated SHR (*p* < 0.05).

### 3.3. Effects of SSa on the DA Levels in Prefrontal Cortex and Striatum

**Figure 3:**
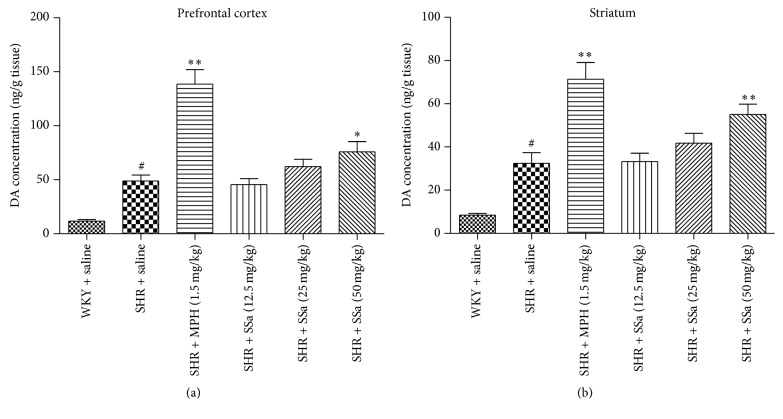
Effects of saikosaponin a or MPH on the DA concentration in PFC (a) and striatum (b). Data are expressed as means ± SEM. *n* = 6, ^#^*p* < 0.01 versus WKY + saline group; ^*∗*^*p* < 0.05, ^*∗∗*^*p* < 0.01 versus SHR + saline group.

To further confirm and understand the therapeutic effects of SSa on ADHD, DA concentration in prefrontal cortex and striatum was measured. As summarized in Figures 3(a) and 3(b), quantitative results revealed that DA concentrations in prefrontal cortex and striatum in SHRs were significantly increased when comparing with the WKY rats (*p* < 0.01). When SSa (25 and 50 mg/kg) or MPH was administrated orally or intraperitoneally, MPH and/or SSa could significantly increase the prefrontal cortex and striatum DA levels in these SHRs (*p* < 0.05 or *p* < 0.01 for each).

### 3.4. Effects of Saikosaponin A on the mRNA Expression of TH, VMAT2, and DAT in PFC

**Figure 4:**
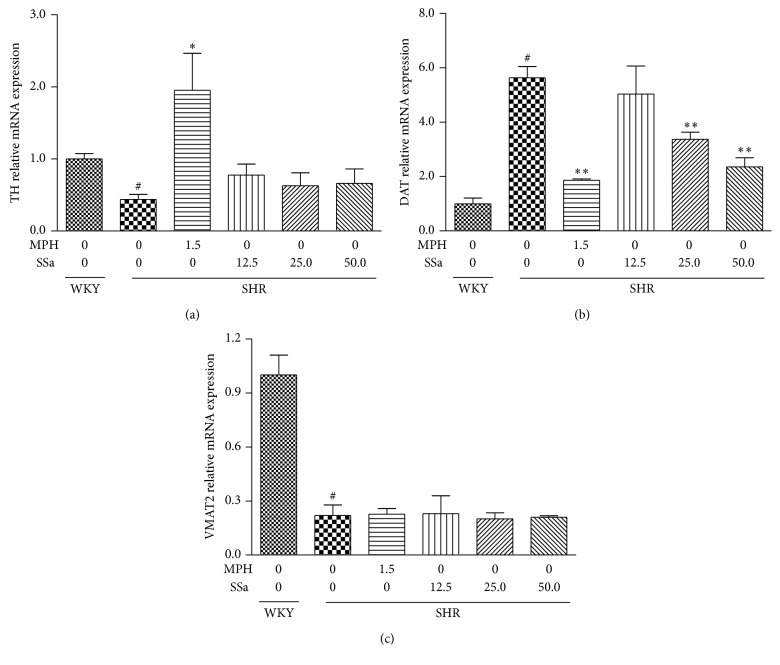
Effects of saikosaponin a or MPH on the mRNA expression of TH (a), DAT (b), and VMAT2 (c) in PFC after a period of 21 days of treatment. Data are expressed as means ± SEM. *n* = 3, ^#^*p* < 0.01 versus WKY + saline group; ^*∗*^*p* < 0.05, ^*∗∗*^*p* < 0.01 versus SHR + saline group.

TH, VMAT2, and DAT have been verified to play an important role in the synthesis, vesicular localization and release, and extracellular persistence of DA. To explore whether SSa treatment was effective to alleviate the ADHD like symptom, the mRNA levels of TH, VMAT2, and DAT in PFC were analyzed. As shown in Figures 4(a), 4(b), and 4(c), SHRs showed a markedly increased mRNA expression of DAT (*p* < 0.01) and decreased mRNA expression of TH and VMAT2 compared with the WKY rats (*p* < 0.01). MPH treatment significantly downregulates the DAT mRNA expression (*p* < 0.01) and upregulates the TH mRNA expression (*p* < 0.01), but with no effects on VMAT2 mRNA expression (*p* > 0.05). SHRs treated with SSa at a dose of 25 mg/kg and 50 mg/kg could significantly reduce the mRNA of DAT as compared with saline-treated SHRs (*p* < 0.01). However, SSa treatment, even at a dose high at 50 mg/kg, possesses little effects on the mRNA expression of TH and VMAT2 when comparing with saline-treated SHRs (*p* > 0.05).

### 3.5. Effects of Saikosaponin A on the Protein Expression of DAT and VMAT2 in PFC

**Figure 5:**
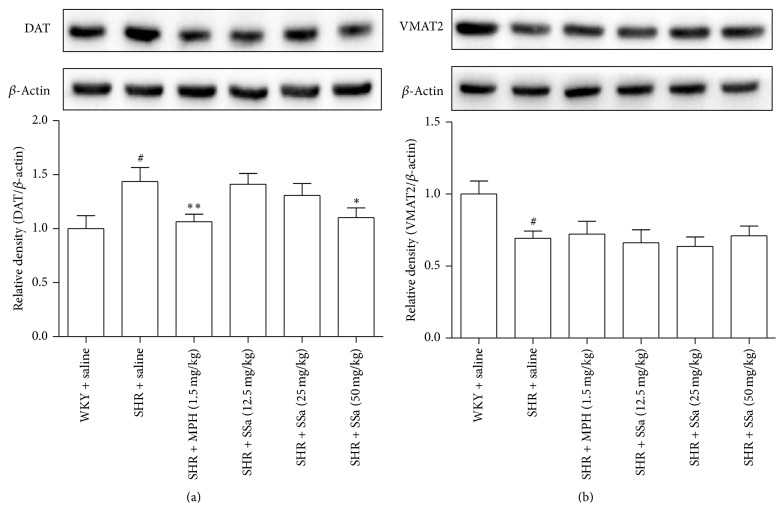
Effects of saikosaponin a on the protein expression of DAT (a) and VMAT2 (b) in PFC. Representative western blots for DAT, VMAT2, or *β*-actin for PFC samples. These data represents means ± SEM. *n* = 6, ^#^*p* < 0.01 versus WKY + saline group; ^*∗*^*p* < 0.05, ^*∗∗*^*p* < 0.01 versus SHR + saline group.

To further determine whether SSa could regulate the protein expression of DAT (Figure 5(a)) and VMAT2 (Figure 5(b)) in PFC, the western blot method was employed. The protein levels of DAT in SHRs were significantly increased as compared to the SHRs (*p* < 0.01), and MPH SSa (50 mg/kg) treatment could reduce the protein levels of DAT in PFC when comparing with the saline-treated SHRs (*p* < 0.01 for MPH, *p* < 0.05 for SSa treatment at a dose of 50 mg/kg). There is no effect of SSa on the protein level of VMAT2 in SHRs, while a downregulated VMAT protein level was observed in MPH treated SHRs when compared to the saline-treated SHRs (*p* < 0.01).

### 3.6. Effects of Saikosaponin A on the Protein Expression of BDNF in PFC

**Figure 6:**
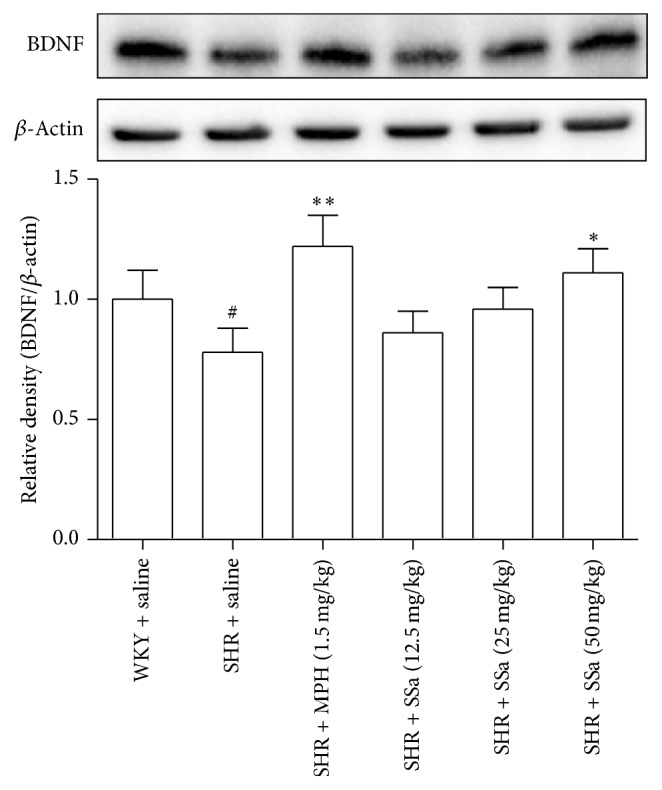
Effects of saikosaponin a on the protein expression of BDNF in PFC. These data represent means ± SEM. *n* = 6, ^#^*p* < 0.01 versus WKY + saline group; ^*∗*^*p* < 0.05, ^*∗∗*^*p* < 0.01 versus SHR + saline group.

To further determine the potential mechanism of the improved spatial learning memory, BDNF level in PFC was studied through the detection of protein contents. As shown in Figure 6, the expressions of BDNF in SHRs were decreased (*p* < 0.01) when comparing with the WKY rats. On the contrary, MPH (1.5 mg/kg) and SSa (50 mg/kg) significantly increase the protein levels of BDNF compared with those in saline-treated SHRs (*p* < 0.01 for MPH, *p* < 0.05 for SSa at a dose of 50 mg/kg).

## 4. Discussion

In this study, we mainly focused on whether SSa could attenuate ADHD symptom and underlying mechanisms on SHRs, an animal model of ADHD. SSa was effective on reducing the hyperactivity and improving the learning memory deficit in SHRs by enhancing the DA bioavailability. This mechanism might be related to reduce the DAT mRNA and an increased BDNF protein expression in brain.

Previous work revealed that ADHD is caused by genetically based abnormalities in those frontal structures of the brain responsible for executive functions [25]. Clinical research also demonstrated that the psychostimulants can reduce activity level and impulsivity, as well as enhance attention-related processes of ADHD children. Several animal models have been developed to study ADHD. Among these animal models, the SHRs are probably the most frequently used animal model for studying the pathological mechanisms of ADHD, due to its similarities with human behavioral responses [26]. In accordance with previous work, our results (Figure 1) also showed that saline-treated SHRs represent hyperactivity symptom, including increased numbers of square crossings, moving distance, and rearing, when comparing with the control rats (WKY rats). After treating with SSa or MPH for 3 weeks, these SHRs exhibited a reduction in locomotor activities. ADHD symptoms might be caused by a disturbed dopamine neurotransmission. Thus we further determine the DA concentration in PFC and striatum, two key brain regions which were involved in mediating cognitive and executive functions, such as sustained attention, working memory, and inhibitory response control [27]. Hypoactivation in frontal cortex has been implicated in ADHD patients [28–30]; also, PFC is identified as the primary target of MPH [31]; that is why we examined PFC region of SHR brain.

Our results (Figures 3(a) and 3(b)) revealed that the DA concentrations after treating with MPH and SSa were significantly increased in those SHRs. These results indicated that SSa may be effective in increasing the extracellular dopamine levels and thereby normalizing the dysregulated dopamine function. Because the DA signaling and distribution are associated with the activity of TH, VMAT-2, and DAT, we investigate the mRNA or protein expression of these factors. TH is a key enzyme and serves as the rate-limiting enzyme in DA production, as it could convert dietary tyrosine to L-dihydroxyphenylalanine (L-DOPA), which may in turn be converted by aromatic amino acid decarboxylase (AADC) to the production of DA. The VMAT2 served as transporting DA from the cytoplasmic space into synaptic vesicles and thus may affect the subsequent neurotransmitter release [12, 13]. DAT could facilitate the reuptake of extracellular DA and ultimately result in degradation of DA.

A decreased TH and VMAT2 mRNA expression and/or activity in PFC or striatum have been observed in the SHRs which were consistent with our date (Figure 4(a)). Also increased DAT mRNA expression and protein levels were observed in the SHRs. Unfortunately, SSa have little effects on the mRNA expression of TH and also cannot upregulate the protein nor mRNA expression of VMAT2. These data indicated that the increased DA levels in brain after treating with the SSa may largely attribute to the decreased DAT mRNA expression and protein levels. Chronic administration of methylphenidate and amphetamine, two frequently used drugs for ADHD, also could target the DAT and decrease DAT density in SHRs, thus possess their beneficial effects, and ameliorate the ADHD symptom. SYN-1A could bind to and regulates the function of DAT and especially participates in the process of membrane fusion and exocytosis [32]. Further study needs to be explored for potential effects of SSa on the SYN-1A and SYN-1A/DAT interaction.

The neurotrophic hypothesis of ADHD suggests that BDNF may play an important role in the development of hyperactivity and learning memory deficit. The hippocampus or PFC have been reported to express a high level of BDNF and may attribute to a normal learning memory in the ADHD [14, 33]. In accordance with previous work, our results also showed that chronic administration of MPH could upregulate the BDNF protein levels in hippocampus or PFC and thus may lead to the improvement of long-term potentiation. Our data demonstrated that MPH could improve the spatial learning memory in the SHRs mainly through the upregulation of the BDNF in the PFC. SSa also could ameliorate the memory deficit in SHRs and increase the protein level of BDNF in PFC of SHRs. Xiaochaihutang, which is an effective TCM prescription for treating depressive disorders, contains Radix bupleuri or SSa also could upregulate the BDNF in specific brain region. Further study was needed to explore whether SSa could act on BDNF's specific high-affinity receptor, tyrosine kinase B (TrkB), or the downstream signal pathway, like PI3K/Akt signaling [34].

## 5. Conclusions

The present study suggests a potential use of saikosaponin a in the treatment of ADHD and finds that its pharmacological effects may be largely associated with the DA availability through the downregulation of DAT and an increase of BDNF in brain. However, further studies were required to study the potential molecular mechanism of these findings.
